# Prognostic and Immune Implications of a Novel Pyroptosis-Related Five-Gene Signature in Breast Cancer

**DOI:** 10.3389/fsurg.2022.837848

**Published:** 2022-05-17

**Authors:** Yuanyuan Zheng, Kainan Wang, Ning Li, Qianran Zhang, Fengxi Chen, Man Li

**Affiliations:** ^1^ Department of Oncology, The Second Hospital of Dalian Medical University, Dalian, China; ^2^ Department of Foreign Language, Dalian Medical University, Dalian, China; ^3^ Department of Breast Diseases, The Second Hospital of Dalian Medical University, Dalian, China

**Keywords:** pyroptosis, breast cancer, tumor immune microenvironment, prognosis, LASSO-Cox regression

## Abstract

**Background:**

Breast cancer (BC) is the most common cancer among women worldwide, with enormous heterogeneity. Pyroptosis has a significant impact on the development and progression of tumors. Nonetheless, the possible correlation between pyroptosis-related genes (PRGs) and the BC immune microenvironment has yet to be investigated.

**Materials and methods:**

In The Cancer Genome Atlas Breast Cancer cohort, 38 PRGs were shown to be significantly different between malignant and non-malignant breast tissues. The 38 PRGs’ consensus clustering grouped 1,089 individuals into two pyroptosis-related (PR) patterns. Using univariate and LASSO-Cox analyses, a PR five-gene predictive signature was constructed based on the differentially expressed genes between two clusters. The tools estimation of stromal and immune cells in malignant tumours using expression data (ESTIMATE), cell type identification by estimating relative subsets Of RNA transcripts (CIBERSORT), and single-sample gene set enrichment analysis (ssGSEA) were used to investigate the BC tumor microenvironment (TME).

**Results:**

In TME, the two PR clusters displayed distinct clinicopathological characteristics, survival outcomes, and immunocyte infiltration features. The developed five-signature model (SEMA3B, IGKC, KLRB1, BIRC3, and PSME2) classified BC patients into two risk groups based on the estimated median risk score. Patients in the low-scoring category had a higher chance of survival and more extensive immunocyte infiltration. An external validation set can yield similar results.

**Conclusion:**

Our data suggest that PRGs have a significant impact on the BC immunological microenvironment. The PR clusters and associated predictive signature stimulate additional research into pyroptosis in order to optimize therapeutic strategies for BC patients and their responses to immune therapy.

## Introduction

Breast cancer (BC), the most common malignancy in women worldwide, is the leading cause of cancer deaths among American women aged 20–59 years, posing a serious danger to women’s health (1). Patients with advanced BC have a poor prognosis due to the high heterogeneity and potential to develop medication resistance (2). Under the multidisciplinary diagnostic and treatment strategy of surgery, chemoradiotherapy, and endocrine and molecular targeted therapy, its death rate has not improved much (3). Traditional pathological risk indicators for recurrence and metastasis in BC, such as tumor size, lymph node metastases, vascular infiltration, and other disease features, are no longer sufficient for assessing the risk of recurrence and metastasis (4). Given the limits of current BC therapies, a new, accurate prognostic model is required to identify high-risk patients and enhance their clinical results.

Pyroptosis is a lytic programmed cell death (PCD) caused by members of the gasdermin family (5). It is distinguished by cellular swelling and vesicle-like protrusions, which are followed by cell membrane rupture and the release of several proinflammatory mediators, including as HMGB14, IL-18, and IL-1, which can elicit inflammatory reactions (5, 6). There are two basic methods for inducing pyroptosis in dying cells: (1) caspase 3 processes GSDME (gasdermin E)-dependent activation and (2) caspase 1/4/5/11 processes GSDMD-dependent activation (7–10). Active caspases cleave the junctional structural domains of GSDME or GSDMD between the N-terminal and C-terminal sections, releasing the activated membrane pore-forming N-terminal domains (6). Swelling, plasma membrane rupture, and the release of immunogenic cellular chemicals are all caused by the huge holes created in the membrane, resulting in enhanced inflammatory reactions, and irreversible cell death (11, 12). Other members of the gasdermin family have comparable N-terminal regions and can also elicit pyroptosis; however, they have received less attention (6). Pyroptosis not only contributes to protection against infections but also plays a vital role in tumorigenesis, metastasis, and drug resistance (13). Recent research found that GSDME expression was elevated in estrogen receptor (ER)-negative cell lines, indicating that it had a role in the carcinogenesis of hormone-inactive BC (14). Another study revealed that overexpression of GSDMB was associated with poor clinical outcomes of human epidermal growth factor receptor 2 (HER2)-targeted therapy for HER2-positive BC (15). The tumor immune microenvironment has already been linked to pyroptosis’s potential anticancer effects. For example, pyroptosis might send out danger signals that encourage the recruitment of antitumor immune cells such as NK and CD8+ T cells (16, 17). GSEME expression can also improve the functionality and number of NK and CD8+ T cells (16). Furthermore, since one property of cancers is the desire to prevent apoptosis, inducing tumor pyroptosis seems to be a very promising treatment strategy (18–20). For example, pyroptosis inducers can collaborate with PD-1/PD-L1 inhibitors to limit tumor growth (18).

In the present study, we discovered 38 pyroptosis-related differentially expressed genes (PR DEGs) between non-cancerous breast tissues and BC. These DEGs classified 1,089 BC samples into two PR clusters with distinct immune microenvironments and survival outcomes. On this premise, we used the LASSO-Cox approach to create a five-gene PR prognostic signature. The estimated risk score can quantify immune infiltration and predict outcomes in BC patients, revealing a possible correlation between pyroptosis and tumor microenvironment (TME).

## Materials and Methods

### Data Collection and Pyroptosis-Related Genes Acquisition

The Cancer Genome Atlas Breast Cancer (TCGA-BRCA) cohort provided RNA sequencing data with associated clinical characteristics and survival statistics for 1,109 BC patients. As an external validation cohort, GSE159956 data encompassing 295 BC samples were obtained from NCBI-GEO. Both the TCGA and GEO databases are open to the public. The current study was carried out in accordance with the standards for data collecting and publishing established by these two databases. Table 1 shows the comprehensive clinicopathological features of these individuals. Following that, 52 PRGs were collected from the Kyoto Encyclopedia of Genes and Genomes (KEGG) and previous reviews and are summarized in Supplementary Table S1 (13, 21–25). We used the “limma” program in the TCGA-BRCA cohort to filter PR DEGs between BC and neighboring non-tumor tissues (*p* < 0.05). Then, using the STRING online database, a protein–protein interaction (PPI) network representing the interactions of PR DEGs was created and downloaded (26).

**Table 1:** Baseline characteristics of BC patients included in this study.

Variables	TCGA cohort	GSE159956 cohort
	*n* = 1,089	*n* = 295
Age		
≤65	770 (70.7%)	NA
>65	319 (29.3%)	NA
Gender		
Female	1077 (98.9%)	NA
Male	12 (1.1%)	NA
T stage		
T1	279 (25.6%)	NA
T2	631 (57.9%)	NA
T3	137 (12.6%)	NA
T4	39 (3.6%)	NA
Unknown	3 (0.3%)	NA
N stage		
N0	513 (47.1%)	151 (51.2%)
N1	360 (33.1%)	N1–N3: 144 (48.8%)
N2	120 (11.0%)	
N3	76 (7.0%)	
Unknown	20 (1.8%)	0
M stage		
M0	906 (83.2%)	194 (65.8%)
M1	22 (2.0%)	101 (34.2%)
Unknown	161 (14.8%)	0
Clinical stage		
Stage I	181 (16.6%)	NA
Stage II	619 (56.8%)	NA
Stage III	246 (22.6%)	NA
Stage IV	20 (1.8%)	NA
Unknown	23 (2.1%)	NA
Estrogen receptor		
Positive	NA	226 (76.6%)
Negative	NA	69 (23.4%)
Status		
Alive	940 (86.3%)	216 (73.2%)
Dead	149 (13.7%)	79 (26.8%)

*NA, not available.*

### Unsupervised Clustering for Breast Cancer Classification

Using “ConsensuClusterPlus” R software, BC samples from the TCGA-BRCA cohort were sorted into discrete PR molecular subtypes based on the expression of 38 PR DEGs. A total of 1,000 iterations were performed to validate the consistency of our categorization.

### Tumor Microenvironment Cell Infiltration

The proportions of 22 immunocytes in BC TME were measured using the CIBERSORT methodology and the “e1071” and “preprocessCore” packages, as well as the LM22 signature, which were retrieved from the CIBERSORT web portal. “ESTIMATE” software was used to compute immune, stromal, and ESTIMATE scores, as well as tumor purity. The “GSVA” program was used to analyze the infiltration scores or activation status of 29 immunological markers in BC samples using single-sample gene set enrichment analysis (ssGSEA).

### Establishment and Validation of a Prognostic Pyroptosis-Related Gene Signature

DEGs between PR clusters were filtered *via* the “limma” R package by setting significance criteria of |log_2_ (fold-change)| > 0.585 (i.e., fold-change (FC) >1.5) and adjusted *p* < 0.05. Univariate Cox analysis based on these DEGs was conducted to acquire prognostic DEGs with a cutoff of *p* < 0.001. Then the LASSO-Cox analysis *via* the “glmnet” R package was employed to build a five-gene prediction model. $\mathrm{Risk}\;\mathrm{score}=\sum_{i}^{5}\beta_{i}P_{i}$ (*β*, risk coefficients; *P*, gene expression level). The estimated median risk score divided BC patients into high- and low-risk subgroups, and Kaplan–Meier analysis was used to assess the survival difference between the two risk groupings. We also used time-dependent receiver operating characteristic (ROC) curves to assess the signature’s reliability. Moreover, Gene Ontology (GO) and KEGG analyses of the DEGs meeting specific criteria of |log_2_FC| > 1 and adjusted *p* < 0.05 between two risk subgroups were conducted *via* the “clusterProfiler” package.

### Nomogram Establishment and Validation

To screen independent predictor factors for overall survival (OS) of BC patients, univariate and multivariate Cox analyses were performed. The risk variables identified in multivariate analysis were then used to create a prediction nomogram to estimate the 3-, 5-, and 10-year OS probability of BC patients in two cohorts. Calibration curves were used to analyze the consistency between anticipated and actual survival results.

### RNA Extraction and Quantitative Real-Time PCR

Total RNA was extracted from breast tissues using Trizol Reagent (Invitrogen, Carlsbad, CA, United States) and reverse-transcribed to produce complementary DNA using the TransScript Uni All-in-One SuperMix reagent (Transgen, Dalian, China). After that, the 2^−ΔΔCT^ approach was utilized to measure gene expression using PerfectStart Green qPCR SuperMix (Transgen) and quantitative real-time PCR (qRT-PCR) analysis. glyceraldehyde-3-phosphate dehydrogenase was used as an internal reference. All the primers are listed in Supplementary Table S2.

### Statistical Analysis

The correlation coefficients were calculated using Spearman’s correlation analysis through the “cor.test” package. The Kaplan–Meier technique with a two-sided log-rank test was used to compare survival chances between subgroups. Dual Cox regression analysis was used to identify independent risk variables for OS. The Wilcox test was used to conduct analyses between two groups, whereas the Kruskal–Wallis test was used to compare several groups. The Pearson *χ*^2^ test was used to compare categorical variables. All statistical analyses were carried out using R v4.1.1. If not specifically stated; *p* < 0.05 represented statistical significance. The workflow diagram is displayed in Supplementary Figure S1.

## Results

### Identification of Pyroptosis-Related Differentially Expressed Genes in TCGA-BRCA Cohort

**Figure 1:**
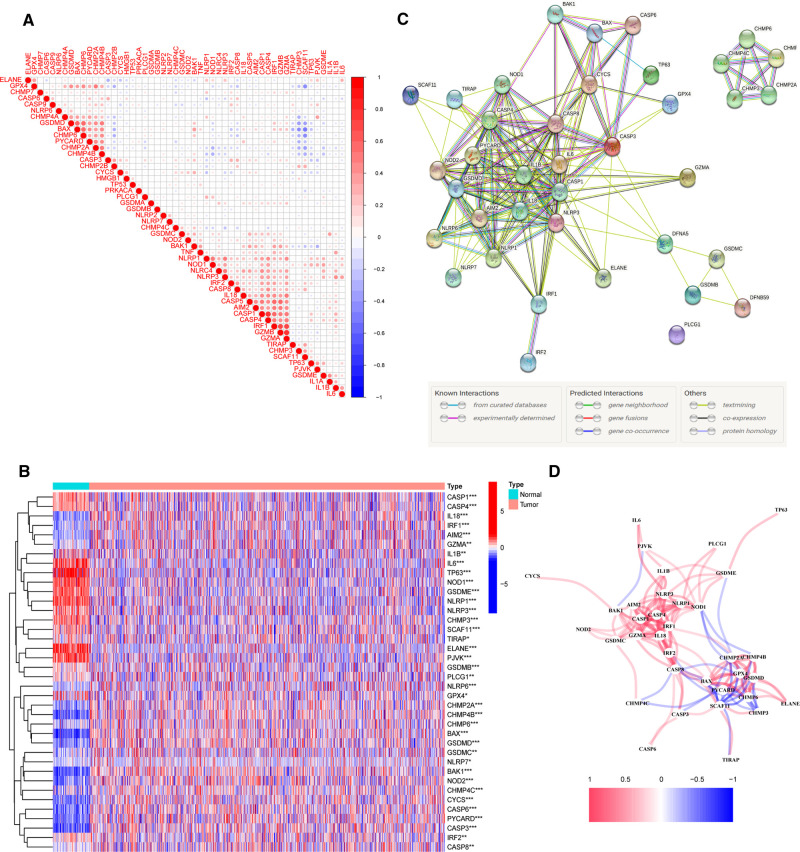
Expression of 38 pyroptosis-related differentially expressed genes PR DEGs in The Cancer Genome Atlas Breast Cancer (TCGA-BRCA) cohort and the interactions among them. (**A**) Correlation analysis among 52 pyroptosis-related (PR) genes. (**B**) Heatmap indicating the expression of 38 PR DEGs between non-cancerous breast tissues and BC. (**C**) Protein–protein interaction network indicating the interactions of 38 PR DEGs. (**D**) Correlation network of 38 PR DEGs. *p < 0.05, **p < 0.01, ***p < 0.001, ns, not significant.

To investigate the relationships between 52 PRGs, a correlation study was performed (blue and red denote negative and positive correlations, respectively) (Figure 1A). We found 38 PR DEGs (all *p*’s < 0.05) by comparing their expression levels in the TCGA-BRCA data across 113 normal and 1,109 tumor tissues. In the BC group, 17 genes among these DEGs (IL6, TP63, ELANE, NLRP1, PJVK, GSDME, NLRP3, NOD1, IL1B, CASP1, CASP4, CHMP3, SCAF11, GPX4, IRF2, TIRAP, and PLCG1) were downregulated, while 21 other genes (CASP8, CHMP6, GSDMB, CHMP4C, CHMP2A, CHMP4B, CYCS, CASP3, IRF1, CASP6, BAK1, GSDMD, GZMA, BAX, IL18, NLRP6, NOD2, PYCARD, AIM2, GSDMC, and NLRP7) were upregulated compared with adjacent breast tissues. The expression and distribution of these DEGs are shown by the heatmap, as shown in Figure 1B. PPI analysis with a minimum needed interaction score of 0.4 was used to study their protein-level interactions further (Figure 1C). Then, in Figure 1D, a gene-level correlation network composed of all DEGs is shown, indicating that the DEGs were strongly linked to one another (red, positive correlations; blue, negative correlations).

### Identification of a Breast Cancer Classification Pattern Mediated by the Pyroptosis-Related Differentially Expressed Genes

**Figure 2:**
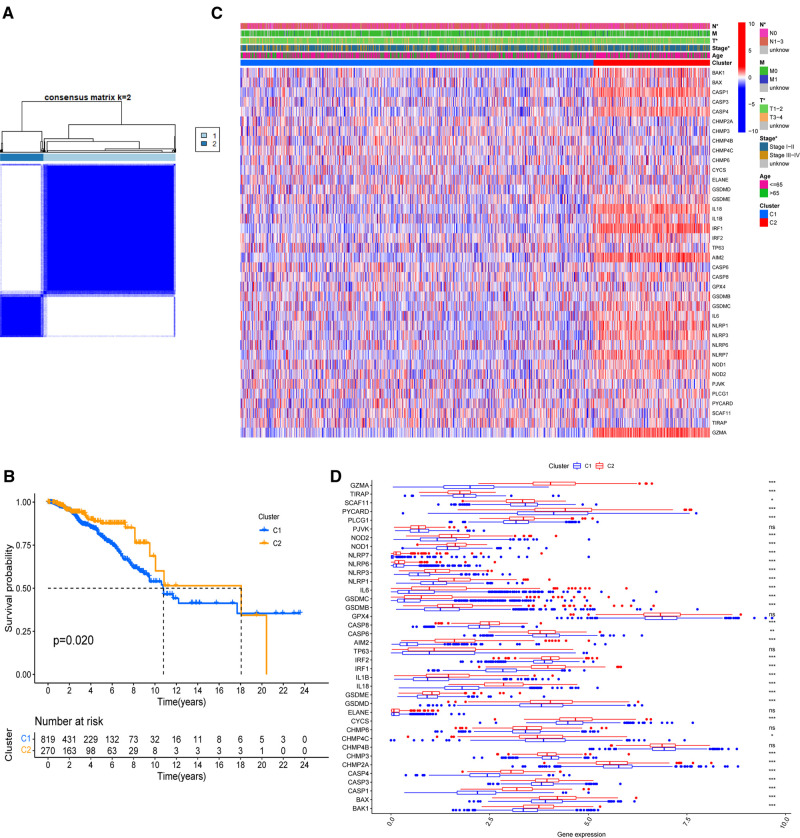
Identification of PR clusters based on 38 PR DEGs. (**A**) Consensus clustering analysis using 38 PR DEGs. All breast cancer (BC) samples were separated into two PR clusters. (**B**) Survival analysis of BC patients for the two PR clusters. (**C**) Heatmap indicating the correlations between two clusters and clinicopathologic characters. (**D**) Expression levels of 38 PR DEGs in two PR clusters. *p < 0.05, **p < 0.01, ***p < 0.001, ns, not significant.

On the basis of the expression of 38 PR DEGs, 1,089 BC patients with full survival data in the TCGA-BRCA cohort were divided into two unique PR patterns using unsupervised consensus clustering analysis (Figure 2A). The intragroup relativity was the highest in this division pattern, and the intergroup relativity was low. There were 819 patients in PR cluster 1 and 270 individuals in PR cluster 2. Through survival analysis, we discovered that cluster 2 had a significantly higher survival advantage than cluster 1 (*p* = 0.02, Figure 2B). Figure 2C shows significant variations in the clinical stage (Stage I–II or III–IV), T stage (T1–2 or T3–4), and N stage (N0 or N1–3) between the two clusters (*p* < 0.05). Furthermore, we discovered that the expression levels of 38 PR DEGs differed in two clusters using expression difference analysis (Figure 2D). The majority of DEGs were more abundant in cluster 2.

### Differences in Tumor Microenvironment Cell Infiltration between Two Pyroptosis-Related Clusters

**Figure 3:**
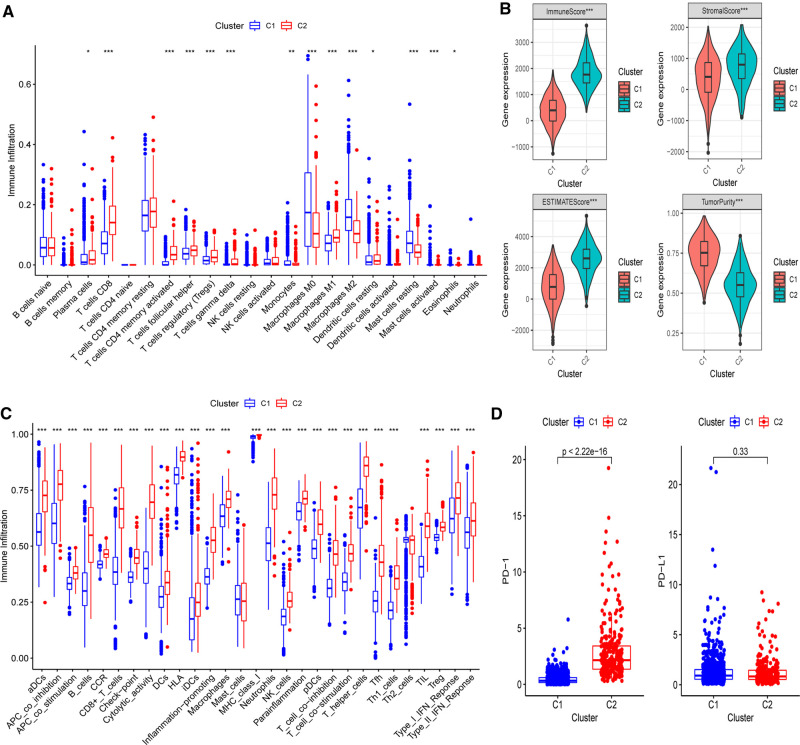
Differences of immuno-infiltrative features between two PR clusters. (**A**) Levels of 22 immunocytes calculated with CIBERSORT in two clusters. (**B**) Levels of ImmuneScore, StromalScore, ESTIMATEScore, and TumorPurtiy calculated with ESTIMATE in the two PR clusters. (**C**) Differences of single-sample gene set enrichment analysis (ssGSEA) scores between two PR clusters. (**D**) Expression of PD-1 and PD-L1 in two PR clusters. *p < 0.05, **p < 0.01, ***p < 0.001, ns, not significant.

Next, the CIBERSORT technique was then used to undertake immunocyte infiltration difference analysis. Monocytes (*p* < 0.01), resting mast cells (*p* < 0.001), and M0 (*p* < 0.001) and M2 macrophages (*p* < 0.001) were found to be over-represented in cluster 1, while activated immunocyte infiltration in TME was abundant in cluster 2, including plasma cells (*p* < 0.05), T-cell regulatory cells (Tregs) (*p* < 0.001), M1 macrophages (*p* < 0.001), as well as activated CD8+ (*p* < 0.001) and CD4+ T cells (*p* < 0.001) (Figure 3A). The data suggested that the proportions of TME cell subsets differed significantly among PR clusters. In addition, we used ESTIMATE to analyze the TME infiltration aspects. The result also showed that cluster 2 had higher immune, stromal, and ESTIMATE scores than cluster 1, indicating that cluster 2’s immune system was completely activated (all *p*’s < 0.001) (Figure 3B and Supplementary Figure S2). Cluster 1 had much higher tumor purity than cluster 2, indicating that cluster 1 may be immune-suppressed (*p* < 0.001). Further assessment of the enrichment levels of immunocytes and immune-associated activities or pathways *via* ssGSEA revealed that, with the exception of Th2 and mast cells (*p* < 0.001), cluster 2 had usually higher scores (Figure 3C and Supplementary Figure S3). The previous data clearly showed that the two clusters had completely distinct TME infiltration and biological molecular characteristics, indicating that pyroptosis had a significant influence on the BC microenvironment. Thus, cluster 1 is an immune-excluded phenotype with high tumor purity and little immune response, whereas cluster 2 is an immune-inflamed phenotype with a significant survival benefit and profuse immune cell infiltration (27). Moreover, since immunotherapy is an emerging treatment option for BC, we then conducted mRNA-level expression difference analysis of PD-1 and PD-L1, the most common immune checkpoints of BC, in two clusters and found a higher level of PD-1 in cluster 2 (*p* < 0.001), implying that immunotherapy is available to patients in this cluster (Figure 3D).

### Establishment and External Validation of a Prognostic Gene Model on the Basis of Pyroptosis-Related Clusters for Breast Cancer Patients

**Figure 4:**
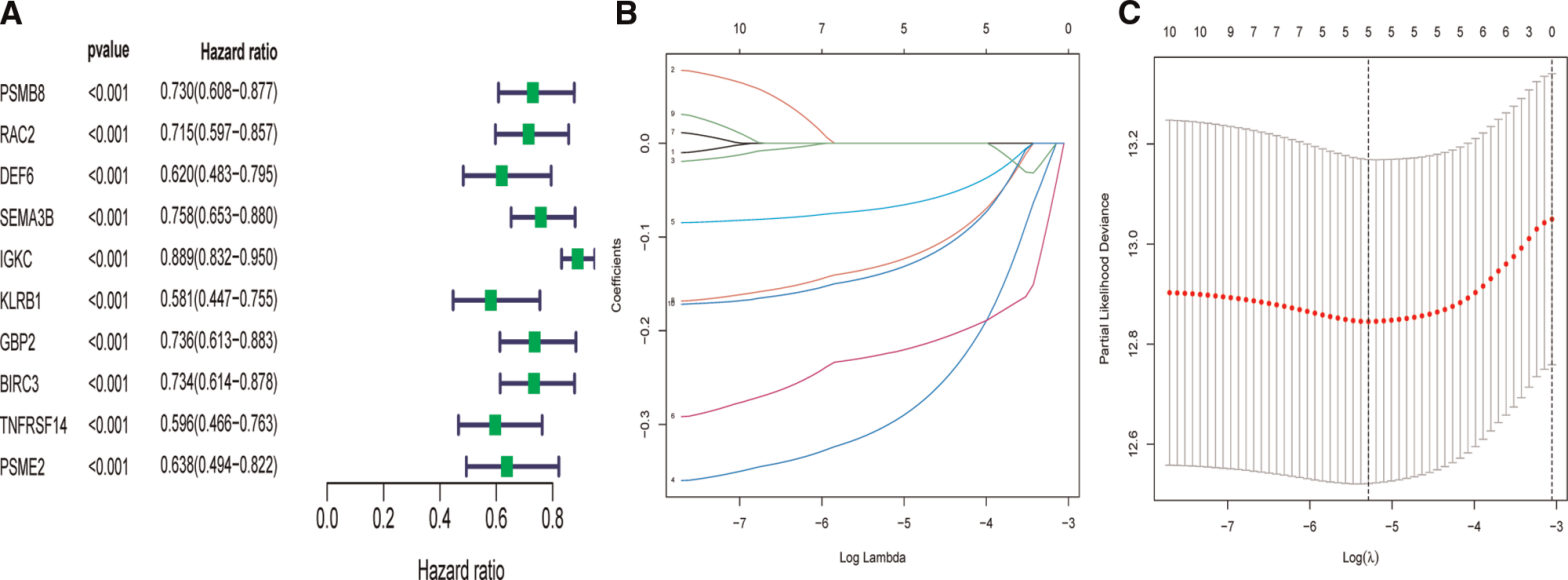
Univariate- and LASSO-Cox analyses based on DEGs between two clusters. (**A**) Univariate Cox analysis of overall survival (OS) and 10 genes with *p* < 0.001. (**B**) LASSO coefficient spectrum of the 10 OS-related genes. (**C**) Cross validation for tuning parameter selection in the LASSO model.

In order to apply the clusters to assist subsequent clinical treatment, we analyzed the differences between the two clusters and established a prognostic gene model. First, we identified considerable DEGs between two clusters that met the requirements of absolute FC > 1.5 and adjusted *p* < 0.05. Next, univariate Cox analysis was used to filter 10 independent prognostic genes with a *p* < 0.001 from the DEGs that were retained for further analysis (Figure 4A). Finally, a five-gene risk signature containing SEMA3B, IGKC, KLRB1, BIRC3, and PSME2 was conducted *via* the LASSO-Cox regression model with the optimal *λ* value (Figures 4B,C). They were all protective genes in BC patients with a hazard ratio (HR) < 1. The risk score can be calculated as follows: risk score = (−0.305 × SEMA3Bexp) + (−0.07 × IGKCexp) + (−0.226 × KLRB1exp) + (−0.131 × BIRC3exp) + (−0.14 × PSME2exp) (Table 2).

**Table 2:** Mean expression and calculated difference value of five genes.

geneID	ConMean	BC.Mean	logFC	*p-*value	FDR	Coef
SEMA3B	7.743299	4.156405	−0.89761	1.79 × 10^−11^	9.72 × 10^−11^	−0.30465
IGKC	288.7621	2010.229	2.799406	5.55 × 10^−65^	1.86 × 10^−63^	−0.0697
KLRB1	1.089098	4.040409	1.891368	5.05 × 10^−84^	2.90 × 10^−82^	−0.22568
BIRC3	3.365708	10.87066	1.691458	8.53 × 10^−75^	3.56 × 10^−73^	−0.13083
PSME2	35.2525	59.46178	0.754237	1.42 × 10^−47^	3.02 × 10^−46^	−0.13956

*ConMean and BC.Mean, mean gene expression in non-cancerous breast tissues and breast cancer tissues, respectively; FDR, false discovery rate; Coef, coefficients in the prognostic model; FC, fold-change.*

**Figure 5:**
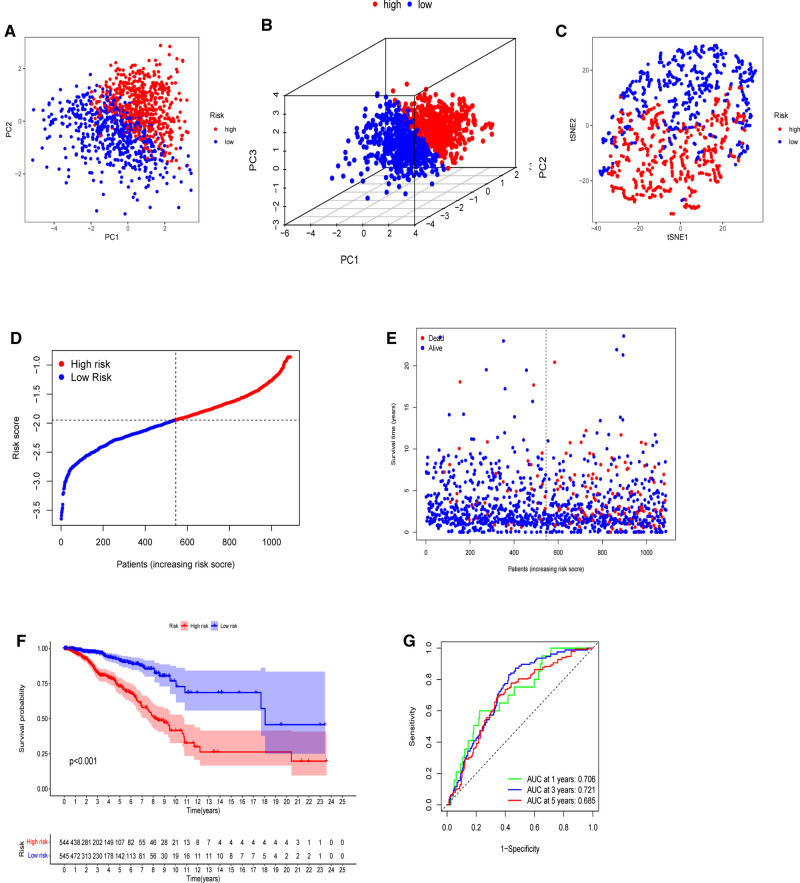
Establishment of a five-gene predictive signature in the TCGA-BRCA cohort. (**A**) Two-dimensional principal component analysis (2D PCA) plot. (**B**) Three-dimensional principal component analysis (3D PCA) plot. (**C**) t-distributed stochastic neighbor embedding (t-SNE) analysis. (**D**) Distribution of calculated risk scores. (**E**) Survival status of BC patients. (**F**) Kaplan–Meier curves of high- and low-risk subgroups. (**G**) Time-dependent receiver operating characteristic (ROC) analysis.

**Figure 6:**
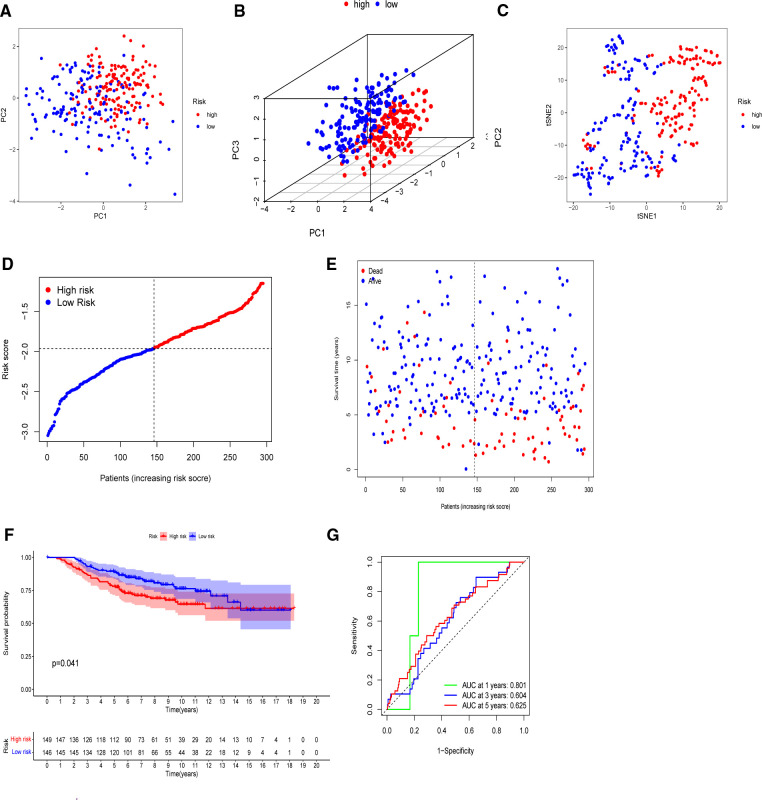
Validation of the five-gene model in the GEO cohort. (**A**) 2D PCA plot. (**B**) 3D PCA plot. (**C**) t-SNE analysis. (**D**) Distribution of calculated risk scores. (**E**) Survival status of BC patients. (**F**) Kaplan–Meier curves of high- and low-risk subgroups. (**G**) Time-dependent ROC analysis.

The model’s delineation effect was validated using two-dimensional principal component analysis (PCA) (Figures 5A, 6A), three-dimensional PCA (Figures 5B, 6B), and t-distributed stochastic neighbor embedding (t-SNE) analysis in both the training (TCGA-BRCA) and independent validation (GSE159956) sets (Figures 5C, 6C). The calculated median score (Figure 5D) was used to divide the 1,089 patients in the TCGA into high-risk and low-risk subgroups. This score was also used to divide the patients in GSE159956 (Figure 6D). In both cohorts, greater mortality and shorter survival duration can be seen in the high-risk subgroup (Figures 5E, 6E). In the TCGA-BRCA cohort, Kaplan–Meier curves of survival analysis revealed that the low-risk group had a greater chance of surviving than the other groups (*p* < 0.001) (Figure 5F), comparable to the validation cohort (*p* = 0.041) (Figure 6F). ROC was applied to determine the reliability of the risk signature. In the TCGA-BRCA cohort, the values of the area under the ROC curve (AUC) at 1, 3, and 5 years were 0.706, 0.721, and 0.685, respectively (Figure 5G). Similarly, the AUC values in the GEO data set at 1, 3, and 5 years were 0.801, 0.604, and 0.625, respectively, suggesting that the prognostic model was accurate (Figure 6G).

### Associations between the Five-Gene Signature and Patients’ Clinicopathological Parameters

**Figure 7:**
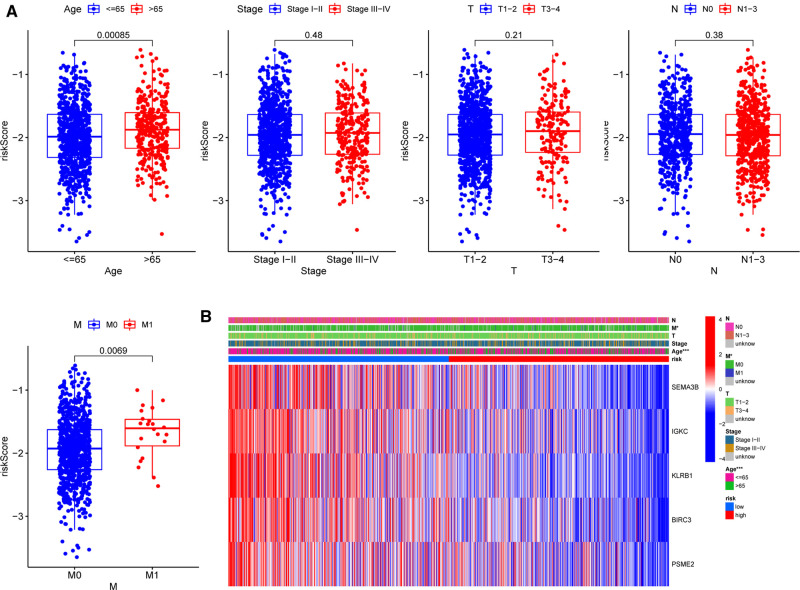
Correlations between signature and clinicopathological parameters. (**A**) Correlations with age, clinical stage, and TNM stage. (**B**) Heatmap indicating the clinicopathologic features of the two risk subgroups. *p < 0.05, **p < 0.01, ***p < 0.001, ns, not significant.

To further assess the clinical implications of our signature, we investigated the relationships between clinical variables and the five-gene signature in the TCGA-BRCA cohort and identified significant variations between M stages and ages (Figure 7A). Patients with M1 stage (*p* = 0.00085) and age above 65 years (*p* = 0.0069) had significantly higher risk scores, demonstrating that our signature was associated with the development of BC. However, the signature was not correlated to the clinical, T, or N phases. The heatmap in Figure 7B depicted the distribution disparities of five genes and clinicopathological variables in two risk groups in an accessible manner. Additionally, we investigated the relationship between the individual gene of the five-gene signature and clinicopathological factors separately (Supplementary Figure S4). In the case of SEMA3B, the level of expression was significantly lower in the T2 stage than that in the T1 stage (*p* = 0.019). Similarly, increased T-stages were associated with lower BIRC3 and KLRB1 expression levels. Interestingly, IGKC and PSME2 expression levels were lower in T3–4 stages than those in T1–2 stages, although there was no significant difference. These findings suggested that the five genes (SEMA3B, BIRC3, KLRB1, IGKC, and PSME2) may play protective roles in BC.

### Roles of Selected Five Signatures in Breast Cancer Prognosis

**Figure 8:**
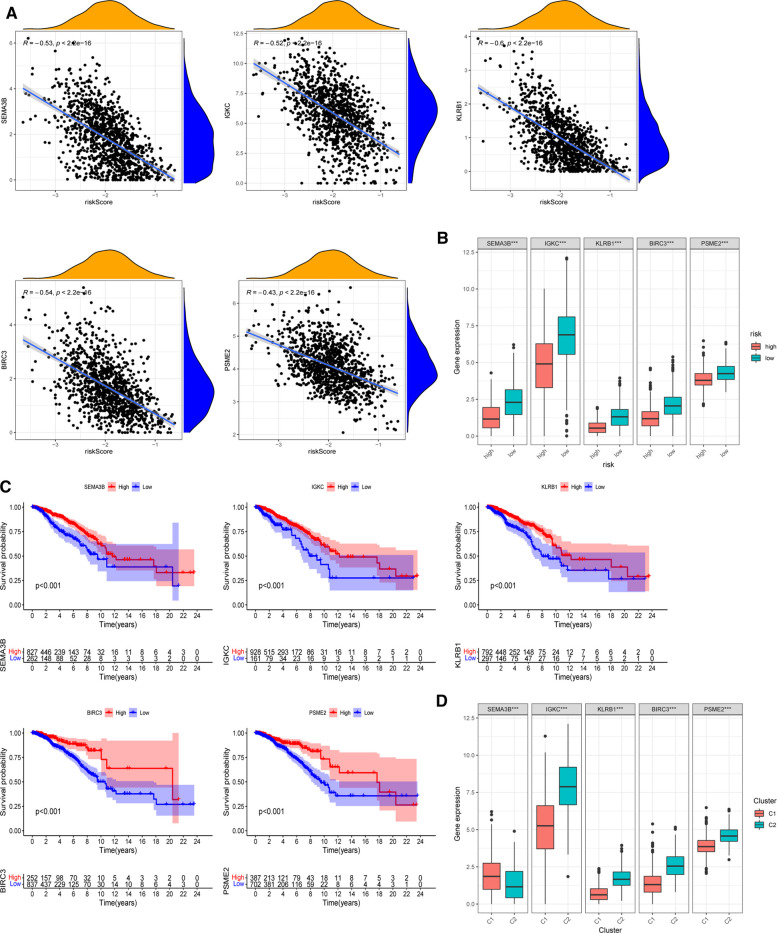
Roles of selected five genes in risk signature and BC prognosis. (**A**) Correlations between risk signature and SEMA3B, IGKC, KLRB1, BIRC3, and PSME2. (**B**) Expression of the five genes in two risk subgroups. (**C**) Kaplan–Meier curves of SEMA3B, IGKC, KLRB1, BIRC3, and PSME2 in the TCGA-BRCA cohort. (**D**) Expression levels of the selected five genes in two PR clusters. *p < 0.05, **p < 0.01, ***p < 0.001, ns, not significant.

The expression of five chosen signatures was shown to be inversely linked with risk ratings using Spearman’s correlation analysis (Figure 8A, *p* < 0.001). Also, the expression of these genes was clearly decreased in the high-risk category (Figure 8B, *p* < 0.001). The findings were consistent with the findings of the Kaplan–Meier survival analysis, which showed that greater gene expression levels of the five chosen genes were associated with a better prognosis for BC patients (Figure 8C, *p* < 0.001). Moreover, the amounts of gene expression varied among two PR clusters (Figure 8D). The considerably greater levels of IGKC, KLRB1, BIRC3, and PSME2 (*p* < 0.001) in immunological cluster 2 may also contribute to its superior OS over cluster 1. Supplementary Figure S5 illustrated the association within these genes. We further analyzed the predictive effects of our five-signature model in different subtypes of BC from the TCGA-BRCA cohort (Supplementary Figure S6). In the luminal (*p* < 0.001) and HER2-overexpressing (*p* = 0.002) subtypes, Kaplan–Meier analysis revealed that the low-risk group had a greater chance of survival than the other group, while there was no significant difference in survival in triple-negative BC.

We also collected seven pairs of BC tissues together with neighboring normal breast tissues from recruited BC patients to determine the clinical relevance of the five-gene signature in BC. Supplementary Figure S7 shows the mRNA-level expression differential analysis of the five prognostic PRGs. Due to tissue heterogeneity and limited patient numbers, dramatically greater or lower expression levels for the same gene can be detected in seven malignant samples compared to normal tissues. We will increase the sample size to confirm whether the results were consistent with the databases.

### Establishment of the Nomogram

**Figure 9:**
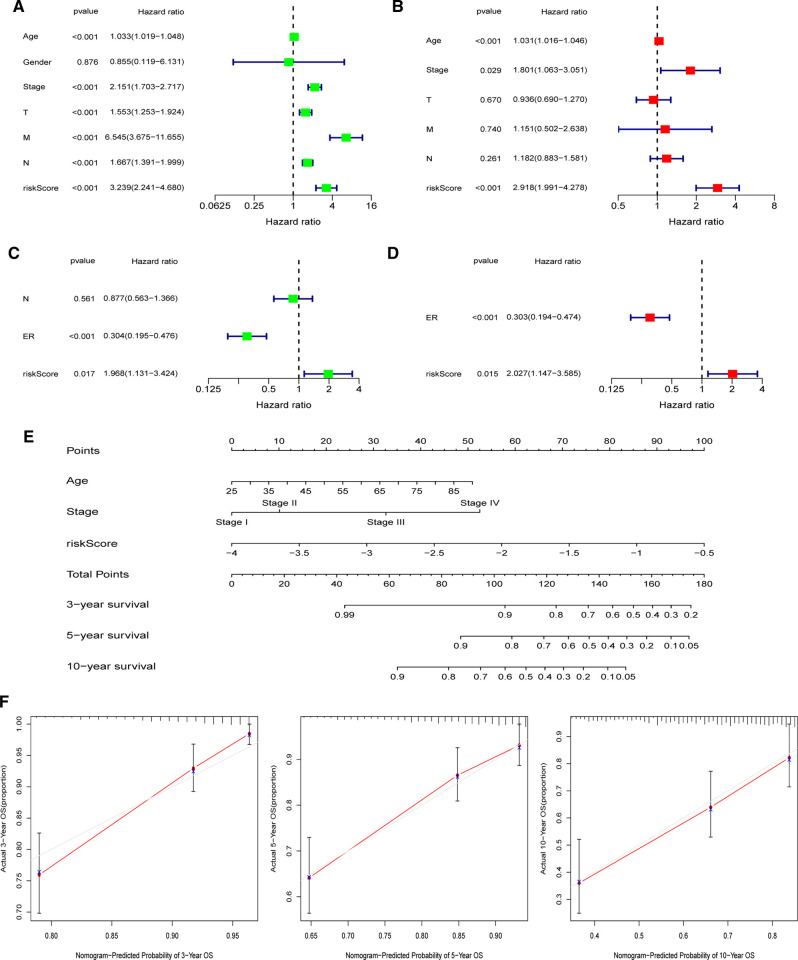
Establishment of the nomogram in the TCGA-BRCA cohort. (**A**) Univariate and (**B**) multivariate COX regression regarding OS in TCGA-BRCA cohort. (**C**) Univariate and (**D**) multivariate COX regression regarding OS in GEO validation cohort. (**E**) Nomogram for 3-, 5-, and 10-year survival prediction in the TCGA-BRCA cohort. (**F**) Calibration curves for assessing the predictive accuracy of the 3-, 5-, and 10-year OS.

In both the TCGA and GEO cohorts, univariate and multivariate Cox regression studies confirmed that the computed risk score might serve as an independent predictor of OS (all *p*’s < 0.05). Meanwhile, multivariate Cox analysis revealed that in the TCGA, clinical stage and age, as well as the ER status in the GEO, were independent predictive markers (Figures 9A–D). In the TCGA-BRCA cohort, the risk score, age, and clinical stage were used to generate a nomogram for 3-, 5-, and 10-year OS predictions (Figure 9E). Calibration curves at corresponding time points of the nomogram demonstrated good consistency between predicted and actual values, reflecting the nomogram’s high accuracy and dependability (Figure 9F). Similarly, in the GEO cohort, a nomogram including the ER status and risk score was created and validated by calibration curves (Supplementary Figure S8).

### Differences of Immune Characteristics between Two Risk Subgroups

**Figure 10:**
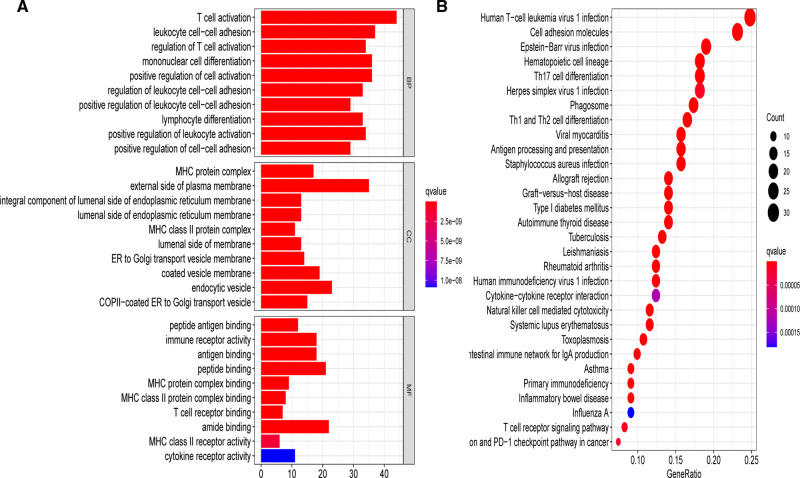
Gene Ontology (GO) and Kyoto Encyclopedia of Genes and Genomes (KEGG) analyses on the basis of DEGs between different risk groups in the TCGA-BRCA cohort. (**A**) Bar plot graph for significant GO enrichment. (**B**) Bubble graph for significant KEGG pathways.

To elucidate the underlying biological roles of the prognostic model, we extracted the DEGs meeting the threshold criteria of false discovery rate (FDR) < 0.05 and |log_2_FC| > 1 between the two risk subgroups. Based on the identified 157 DEGs in the TCGA-BRCA cohort, we performed GO and KEGG enrichment analyses, which suggested that these DEGs were mainly involved in T cell activation and leukocyte cell–cell adhesion biological processes, as well as the human T-cell leukemia virus 1 infection pathway (Figure 10).

**Figure 11:**
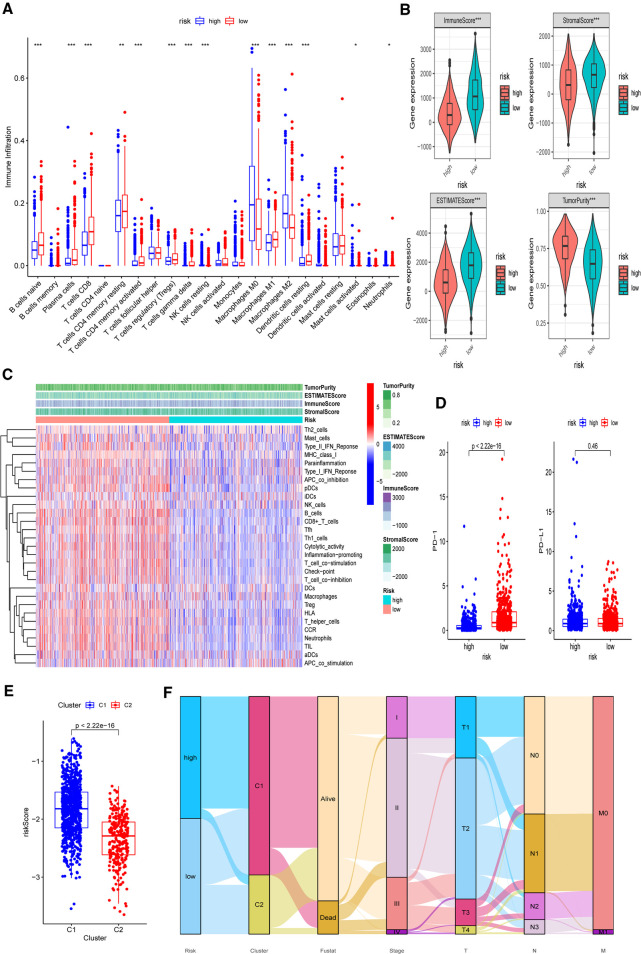
Differences of immuno-infiltrative features between the two risk subgroups. (**A**) Levels of 22 immunocytes calculated with CIBERSORT in two risk subgroups. (**B**) Levels of ImmuneScore, StromalScore, ESTIMATEScore, and TumorPurtiy calculated with ESTIMATE in the two risk subgroups. (**C**) Heatmap indicating the differences in ssGSEA scores between two risk subgroups. (**D**) Expression levels of PD-1 and PD-L1 in two risk subgroups. (**E**) Associations between calculated risk scores and PR clusters. (**F**) Alluvial diagram displaying the changes in the risk score, PR clusters, survival status, cinical stage, and TNM stage in TCGA-BRCA cohort. *p < 0.05, **p < 0.01, ***p < 0.001, ns, not significant.

Based on the functional analyses, the differences in immune infiltration between the two risk groups were examined. In the low-risk group, there is a greater infiltration of activated immune cells, including naive B cells, Tregs, M1 macrophages, and activated CD8+ and CD4+ T cells (*p* < 0.001) (Figure 11A). Additionally, the low-risk group had higher immune, stromal, and ESTIMATE scores (*p* < 0.001) than the high-risk group (Figure 11B). Then, using CIBERSORT data, we performed correlation studies between the risk score and the infiltration levels of 22 different types of immunocytes. The findings indicated that 15 immune cells were directly related to the risk score, with both positive and negative correlations (Supplementary Figure S9). In both the TCGA-BRCA and GEO cohorts, the ssGSEA approach yielded similar results. Using ssGSEA, we observed that the low-risk category had universally greater immunocyte infiltration and immune pathway activation (Figure 11C and Supplementary Figure S10). Furthermore, the mRNA expression level of PD-1 was clearly elevated in low-risk patients, indicating that they had a greater chance of receiving immunotherapy (Figure 11D, *p* < 0.001). We further investigated the associations between five chosen genes and TME by comparing immunocyte infiltration levels between each gene’s high and low expression subgroups (Supplementary Figure S11). Correlation studies between immunocytes and gene expression were also carried out (Supplementary Figure S12). We also discovered a strong association between these genes and activated immune cells, such as naive B cells, M1 macrophages, activated CD4+ memory, and CD8+ T cells (*p* < 0.05).

Finally, we investigated the difference in estimated risk ratings between two PR clusters (Figure 11E). Cluster 2 exhibited a much lower risk score, which corresponded to its demonstrated survival advantage and substantial immunocyte infiltration (*p* <  0.001). The distributions of clinicopathological characteristics in distinct risk groups and clusters were shown in an alluvial diagram (Figure 11F). High-risk patients were found to be mostly clustered in cluster 1.

## Discussion

We initially investigated the expression of 52 well-defined PRGs in malignant and non-malignant tissues and found that 38 of them were differentially expressed. Based on these DEGs, we revealed two distinct PR clusters *via* consensus clustering analysis. Cluster 1 was an immune-excluded phenotype with high tumor purity and little immune response; cluster 2 was an immune-inflamed phenotype with a significant survival benefit and profuse immune cell infiltration. Furthermore, there were substantial disparities in the distribution of the clinical stage, T stage, and N stage between these two groups. Then, we created a unique five-gene risk signature based on the DEGs between two PR clusters and validated its predictive value using an external GEO data set. The investigation of immune infiltration features in two risk categories revealed that immune activity in high-risk BCs was generally lowered. Furthermore, we created predictive nomograms by integrating risk scores and clinical risk indicators in the hopes of guiding the following treatment for BC patients. Although a PR risk profile for BC has previously been developed, the study only used 33 PRGs to undertake consensus clustering analysis and design a predictive model (28). More PRGs are involved in our investigation, leading to the discovery of more underlying molecular markers.

Pyroptosis, a novel PCD process identified after autophagy and apoptosis, is thought to have two roles in tumor development. On the one hand, by releasing proinflammatory mediators and eliciting inflammatory responses, pyroptosis can promote carcinogenesis and contribute to chemoresistance (29–31). On the other hand, pyroptosis can eliminate abnormal cells and slow tumor growth by serving as a kind of PCD (29, 32). In BC, the connection between PRGs and patient survival outcomes is mostly unclear. In both the TCGA-BRCA and GEO cohorts, our PR prognostic model was shown to have independent prognostic value. It included SEMA3B, IGKC, KLRB1, BIRC3, and PSME2. All of these genes were shown to be protective genes in our study, indicating a favorable prognosis for BC patients. The TCGA database revealed that these genes have decreased expression levels in higher T-stages, confirming the aforementioned findings. SEMA3B (Semaphorin 3B), a tumor-suppressing axon guidance factor, has been shown to induce apoptosis in a range of cancers, including the lung, renal, and gastric cancer (33–37). It was also an angiogenesis inhibitor, and its antiangiogenic effects could be avoided by increasing the expression of furin-like proprotein convertases (38). Suppression of microRNA-221 in glioma cells inhibited cell proliferation and invasion *via* decreasing SEMA3B (39). SEMA3B was discovered to be a unique GATA3 downstream target gene in mammary epithelial cells, suppressing BC growth and metastasis (36). Moreover, it can also promote prometastatic TME in lung cancers by increasing IL-8 production and macrophage recruitment (40). The current study found a strong correlation between SEMA3B expression and the degree of infiltration of numerous immunocytes in BC TME. Immunoglobulin kappa C (IGKC) is an essential component of humoral immunity. Previous research has shown that it can predict the success of neoadjuvant chemotherapy in BC patients (41, 42). IGKC has been shown to be an independent protective factor for OS and metastasis-free survival in node-negative BC (42–44). In our research, IGKC was a good prognostic gene in both node-positive and node-negative BC. KLRB1 (killer cell lectin-like receptor B1) is a surface marker produced by the vast majority of T cell subsets that can signify innate immunity (45, 46). Several studies (45, 47, 48) found that it had a possible prognostic value for good survival in non-uterine leiomyosarcoma and BC, which was consistent with our results that KLRB1 had a protective function in BC. BIRC3 (baculoviral IAP repeat-containing 3) belongs to the apoptosis inhibitor family (49). Many studies have shown that it has antiapoptotic and prosurvival properties (50). By boosting NF-B nuclear translocation, BIRC3 can predict the course of chronic lymphocytic leukemia (CLL) and characterize therapy sensitivity (50, 51). By boosting NF-B nuclear translocation, BIRC3 can predict the course of CLL and characterize therapy sensitivity (50). Additionally, microRNA-124 can target BIRC3 and alter the NF-κB pathway to decrease HCC growth (52). In BC, esophageal adenocarcinoma, and glioblastoma, BIRC3 is associated with treatment resistance (53–55). For example, IL-1 can stimulate BIRC3 upregulation and give chemoresistance to doxorubicin in BC cells (53). Our PR gene signature also revealed that it plays a function in pyroptosis. PSME2 (proteasome activator subunit 2) participates in protein hydrolysis by degrading damaged proteins. Its expression is increased in gastric and renal malignancies while decreasing in lung cancer (56–58). PSME2 overexpression has been linked to clear cell renal cell carcinoma invasion by blocking BNIP3-mediated autophagy (59). Previous studies on the role of PSME2 in tumorigenesis showed contradictory results. It was found to have pro- and anticancer effects in two investigations on gastric cancer (60, 61). Thongwatchara et al. found that overexpression of PSME2 increased tumorigenicity in BC (62). However, PSME2 was found to be a predictive protective gene in our study. The contradictory results underscore the need for more research on this gene. Besides, the gene encodes protein PA28, which can modulate gastric cancer cell invasion by regulating the level of chloride intracellular channel 1 (63). It may also activate proteasomes, influencing antigen processing (64).

Previous studies have explored the underlying modulation between pyroptosis and tumor immunity. Young et al. implicated that effector T cells can harness GSDMD to form pores in cell membranes (65). GSDMD was shown to be engaged in cytotoxic T lymphocyte (CTL)-mediated cell death in lung squamous carcinoma, and its absence weakens the CTL-killing potential (66). In the research of Zhou et al., NK cells and CD8+ T cells were reported to induce pyroptosis and trigger tumor clearance by activating the GSDMB-granzyme A axis (67). Pyroptotic macrophages can increase the cytotoxicity of NK cells in hepatocellular carcinoma by producing CCL5 and IL-18 and thus kill more tumor cells (68). Wang et al. also observed that immune checkpoint inhibitors could only eradicate cold tumor cells when pyroptosis was present (17). Analyses of TME immune infiltration, immunological checkpoints, and clinicopathological characteristics in the current study revealed significant differences between the two risk groups, suggesting the importance of our PR risk profile in immunotherapy. Pyroptosis combined with immunotherapy may be a very promising therapeutic direction for the improvement of prognosis. Besides, the ssGSEA scores of most immunocytes and pathways were decreased in high-risk BCs. Thus, we hypothesized that overall immunological dysfunction and immune infiltration imbalance might contribute to high-risk BCs’ poor survival outcomes.

There exist several limitations in the current research. First, our PR prognostic model was based on retrospective data extracted from public databases. We will further testify its clinical practicability in different molecular subtypes using prospective multicenter data. Second, we merely utilized PRGs to establish the predictive model so that other predominant prognostic signatures might be precluded. Moreover, further experimental validation is warranted to investigate the relativity between our model and immune activity.

## Conclusions

In conclusion, our findings revealed a strong correlation between pyroptosis and the development of BC. The broad influence of PRGs on the tumor immunological microenvironment was also proved. In addition, we developed a new PR prognostic signature and verified its independent predictive significance for OS. The potential relationship between PRGs and tumor immunity in BC deserves further exploration.

## Data Availability

The raw data supporting the conclusions of this article will be made available by the authors without undue reservation.
