# Supplementary material for: Prognostic and Immune Implications of a Novel Pyroptosis-Related Five-Gene Signature in Breast Cancer
Source: Front Surg. 2022 May 17;9:837848. doi: 10.3389/fsurg.2022.837848 (PMC9152226; doi:10.3389/fsurg.2022.837848)
Supplement: Supplementary file 1 [file Data_Sheet_1_v2.doc]

**Figure S1.** The workflow diagram of data analysis.

**Figure S2.** Heatmap indicating the different levels of ImmuneScore, StromalScore, ESTIMATEScore and TumorPurtiy between two pyroptosis-related clusters. *P<0.05, **P<0.01, ***P<0.001, ns not significant.

**Figure S3.** Heatmap indicating the differences of ssGSEA scores between two pyroptosis-related clusters. *P<0.05, **P<0.01, ***P<0.001, ns not significant.

**Figure S4.** The comparison of the five genes’ expression levels in different T-stages from the TCGA database. *P<0.05, **P<0.01, ***P<0.001, ns not significant.

**Figure S5.** Correlations within selected five genes.

**Figure S6.** The Kaplan-Meier analyses of our five-signature model in various subtypes of BC from the TCGA cohort.

**Figure S7.** The relative expression levels of the five genes in normal and BC tissues. *P<0.05, **P<0.01, ***P<0.001, ****P<0.0001.

**Figure S8.** Construction of a nomogram in GEO validation cohort. (A) Nomogram for 3-, 5- and 10-year survival prediction in GEO validation cohort. (B) Calibration curves for assessing the predictive accuracy of the 3-, 5- and 10-year OS.

**Figure S9.** Correlation analyses between calculated risk scores and infiltration degrees of immune cells from CIBERSORT.

**Figure S10.** (A) The enrichment scores of 16 immunocytes and (B) 13 immune-associated pathways in TCGA-BRCA cohort. (C) The enrichment degrees of immunocytes and (D) immune-associated pathways in GEO cohort. *P<0.05, **P<0.01, ***P<0.001, ns not significant.

**Figure S11.** Differences in immunocyte infiltration degrees between high-expression and low-expression subgroups of 5 selected genes (green: low expression group; red: high expression group).

**Figure S12.** Correlation analyses between immunocytes and gene expression.

**Table S1. 52 pyroptosis-related genes in this study.**

**Table S2. Primers design and their sequences.**
