# Supplementary material for: Prognostic and Immune Implications of a Novel Pyroptosis-Related Five-Gene Signature in Breast Cancer
Source: Front Surg. 2022 May 17;9:837848. doi: 10.3389/fsurg.2022.837848 (PMC9152226; doi:10.3389/fsurg.2022.837848)
Supplement: Supplementary file 2 [file Table_3_v1.doc]

**Table S1. 52 pyroptosis-related genes in this study.**

| BAK1 | CHMP7 | TP63 | NLRP3 |
| --- | --- | --- | --- |
| BAX | CYCS | AIM2 | NLRP6 |
| CASP1 | ELANE | CASP6 | NLRP7 |
| CASP3 | GSDMD | CASP8 | NOD1 |
| CASP4 | GSDME | CASP9 | NOD2 |
| CASP5 | GZMB | GPX4 | PJVK |
| CHMP2A | HMGB1 | GSDMA | PLCG1 |
| CHMP2B | IL18 | GSDMB | PRKACA |
| CHMP3 | IL1A | GSDMC | PYCARD |
| CHMP4A | IL1B | IL6 | SCAF11 |
| CHMP4B | IRF1 | NLRC4 | TIRAP |
| CHMP4C | IRF2 | NLRP1 | TNF |
| CHMP6 | TP53 | NLRP2 | GZMA |
