# Supplementary material for: Prognostic and Immune Implications of a Novel Pyroptosis-Related Five-Gene Signature in Breast Cancer
Source: Front Surg. 2022 May 17;9:837848. doi: 10.3389/fsurg.2022.837848 (PMC9152226; doi:10.3389/fsurg.2022.837848)
Supplement: Supplementary file 3 [file Table_4_v1.doc]

| **No.** | **Gene Primer** | **Base sequence (5'to 3')** | **Base number** | **Purification method** |
| --- | --- | --- | --- | --- |
| 1 | GAPDH-F1 | GGAGCGAGATCCCTCCAAAAT | 21 | PAGE |
| 2 | GAPDH-R1 | GGCTGTTGTCATACTTCTCATGG | 23 | PAGE |
| 3 | SEMA3B-F1 | ACGTCCAAGTCTCCGAACAGA | 21 | PAGE |
| 4 | SEMA3B-R1 | GCCGTCTCACGAAAGAAGAAGT | 22 | PAGE |
| 5 | IGKC-F1 | GATCTGGGACAGAATTCACTCTCA | 24 | PAGE |
| 6 | IGKC-R1 | GCCGAACGTCCAAGGGTAA | 19 | PAGE |
| 7 | KLRB1-F1 | CCCTTGGAATAACAGTCTAGCTG | 23 | PAGE |
| 8 | KLRB1-R1 | TTGTCACGTATCAGGTTCTGTG | 22 | PAGE |
| 9 | BIRC3-F1 | TTTCCGTGGCTCTTATTCAAACT | 23 | PAGE |
| 10 | BIRC3-R1 | GCACAGTGGTAGGAACTTCTCAT | 23 | PAGE |
| 11 | PSME2-F1 | GCAAGAGGACTCCCTCAATGT | 21 | PAGE |
| 12 | PSME2-R1 | CTTCTGGCTTAACCAGGGCA | 20 | PAGE |

**Table S2. Primers design and their sequences.**
